# A randomized controlled trial of sucrose and/or pacifier as analgesia for infants receiving venipuncture in a pediatric emergency department

**DOI:** 10.1186/1471-2431-7-27

**Published:** 2007-07-18

**Authors:** Sarah J Curtis, Hsing Jou, Samina Ali, Ben Vandermeer, Terry Klassen

**Affiliations:** 1 Department of Pediatrics, University of Alberta, Edmonton, Alberta, Canada

## Abstract

**Background:**

Although sucrose has been accepted as an effective analgesic agent for procedural pain in neonates, previous studies are largely in the NICU population using the procedure of heel lance. This is the first report of the effect of sucrose, pacifier or the combination thereof for the procedural pain of venipuncture in infants in the pediatric emergency department population.

**Methods:**

The study design was a double (sucrose) and single blind (pacifier), placebo-controlled randomized trial – factorial design carried out in a pediatric emergency department. The study population was infants, aged 0 – 6 months. Eighty-four patients were randomly assigned to one of four groups: a) sucrose b) sucrose & pacifier c) control d) control & pacifier. Each child received 2 ml of either 44% sucrose or sterile water, by mouth. The primary outcome measure: FLACC pain scale score change from baseline. Secondary outcome measures: crying time and heart rate change from baseline.

**Results:**

Sucrose did not significantly reduce the FLACC score, crying time or heart rate. However sub-group analysis revealed that sucrose had a much greater effect in the younger groups. Pacifier use reduced FLACC score (not statistically significant), crying times (statistically significant) but not heart rate. Subgroup analysis revealed a mean crying time difference of 76.52 seconds (p < 0.0171) (0–1 month) and 123.9 seconds (p < 0.0029) (1–3 month). For subgroup age > 3 months pacifier did not have any significant effect on crying time. Age adjusted regression analysis revealed that both sucrose and pacifier had significant effects on crying time. Crying time increased with both increasing age and increasing gestational age.

**Conclusion:**

Pacifiers are inexpensive, effective analgesics and are easy to use in the PED for venipuncture in infants aged 0–3 months. The benefits of sucrose alone as an analgesic require further investigation in the older infant, but sucrose does appear to provide additional benefit when used with a pacifier in this age group.

**Trial registration:**

Current Controlled Trials ISRCTN15819627

## Background

To facilitate proper assessment, diagnosis and management many children must undergo painful procedures such as venipuncture for blood work or treatment, making the emergency department an ideal location to evaluate effective methods of pain control. Research suggests that prompt and accurate recognition and treatment of pain in young infants is important for their immediate comfort and for their best possible lifelong development [1,2]. Despite the recent interest in pediatric pain assessment, prevention and treatment, many children are still not adequately treated to alleviate pain [[1,3], and [4]].

The ideal analgesic for procedural pain in the emergency department should have quick onset, be effective, and have no side effects. Sucrose has been extensively studied as analgesia for short procedures such as heel lance in neonates. A Cochrane systematic review concluded that sucrose is safe and effective in reducing procedural pain from single short procedural events in neonates [5].

This taste-induced analgesia is thought to be mediated by endogenous opiod mechanisms [[6,7], and [8]] although has been questioned in other papers. Gradin et al have demonstrated that administration of an opiod antagonist to newborns did not reduce the pain relieving effect of oral glucose [9]. This contradicts findings in previous animal studies. Also, Eriksson et al showed that tolerance did not develop in neonates who were given repeated doses of glucose [10]. Infants receiving immunizations up to 12 months of age had similar findings [11]. Other theories for the analgesic actions of sucrose are through non-opiod endogenous pain inhibiting systems, activation of the pleasure center with dopamine release and initiation of the sucking response.

The effect of non-nutritive sucking using pacifiers has also been studied in neonates. Sucking is thought to trigger release of serotonin, which may modify the perception of pain. In general the magnitude of the decrease in pain is greater when sucking and sucrose are combined than with sucking alone [7].

From the neonatal literature, which most frequently examined pain responses to heel lance, it seems that sucrose is a safe, easy-to-administer, inexpensive and effective analgesic for short painful procedures. A growing number of studies, looking at infants undergoing immunizations, suggest that this analgesic effect may indeed extend past the neonatal period into infancy [11-16]. Nevertheless, the upper limit of this effect is unknown in terms of age and appropriate sucrose strength. Also, the analgesic effect of non-nutritive sucking for infants older than one month has not been previously studied.

We wished to know if the analgesic effect of sucrose or pacifier holds true for neonates and young infants in the emergency department, particularly for the procedure of venipuncture, which unlike heel lance and intramuscular injection, is commonly performed in that setting. Patients frequenting the emergency department generally differ in physiology and pathology from that of neonates in intensive care units where the majority of previous studies were carried out. We anticipated that this study would provide direction as to whether the use of sucrose, plus or minus pacifiers, as analgesia for venipuncture is useful for infants undergoing assessment in pediatric emergency departments.

## Methods

### Location

This study took place at the Pediatric Emergency Department at the Stollery Children's Hospital in Edmonton, Alberta, Canada. The Stollery Children's Hospital houses the only specialized pediatric emergency in central and northern Alberta and has one of the largest catchment areas in North America with its referral base of more than 1.7 million. The PED sees 50 to 60 children per day with annual figures totaling just over 20,000 patients. Approximately 15 per cent of these emergency patients require admission to hospital.

### Protocol

**Figure 1:**
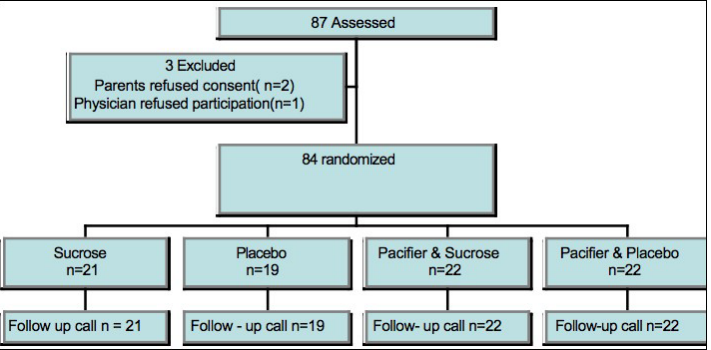
Study Flow Diagram.

This study received approval from the hospital's institutional ethics review board, the department of pediatrics and from the division of emergency medicine. Figure 1 outlines the study flow.

### Study population

All infants up to 6 months corrected age that required venipuncture as part of their emergency department management were eligible for the study. Participants were required to have had nothing by mouth for 5 minutes prior to study commencement. Previous animal and human studies have shown that sucrose analgesia lasts for up to five minutes. Exclusion criteria included any infant deemed critically ill at the discretion of the attending physician, fructose intolerance, and EMLA application at site of venipuncture.

### Blinded randomization

The subjects were randomized to the treatment groups using computer-generated block randomization organized by the hospital research pharmacist. Each pre-prepared syringe holding either sucrose or sterile water was labeled by numbers 1–84 and was indistinguishable by color, smell and flow during administration. The study syringes were placed in a sealed package and stored in a fridge in an area of the ED to which only the ED nurses had access. After written informed consent was obtained, the study nurse obtained the next labeled syringe. The number on the syringe was recorded with the patient's data and on a separate list containing the patient's name, which was kept separate from the data.

The research pharmacist held the numbered code list containing the identity of the solution used in each syringe until the study had officially ended and data analysis was completed. It was then released to the primary author and statistician so that correct identification of groups could occur. Thus throughout the study all of the researchers, outcome assessors, subjects and statistician were blinded to the identities of the solutions.

Pacifier use could not be blinded to the research nurse or parent since visualization of the face was required to score the primary outcome measure. The addition of a second research nurse to stand behind a curtain to take crying time measurements would have added considerable expense to the study and was not feasible. Random assignment of pacifier use was again computer generated by the pharmacist, so that each numbered syringe included instructions to use or omit pacifier. The researchers and statistician were blinded as to which patients had used pacifiers until after completion of analysis.

### Outcome measurements

The pain related to venipuncture was primarily measured using the Face, Legs, Activity, Cry, and Consolability Pain Scale (FLACC) [17-19]. This scale was validated by Merkel et al for measurement of pain in preverbal or cognitively impaired children, and is used by the pediatric pain service at SCH. The FLACC tool assesses changes in the above five categories of behavior, rating each on a scale of 0–2 (Table 1). Ten is the maximum score indicating severe pain and a score < 2 generally indicates absence of pain. FLACC scores were assessed before procedure and after venipuncture and change from baseline was our outcome measure. Interrater reliability for this scale has been demonstrated to be acceptable as kappa values for each of the five categories range between 0.52 and 0.82. It is generally acknowledged that interrater reliability coefficients over 0.41 demonstrate acceptable agreement between users [19]. Several different research nurses were trained by our research nurse coordinator in performing FLACC scores and other details related to outcome assessment prior to each of the study periods. One refresher session was offered throughout each study period also to ensure skills remained consistent.

**Table 1:** FLACC pain scale

**Categories**	**Scoring**		
	0	1	2

**Face**	No particular expression or smile	Occasional grimace or frown, withdrawn, disinterested.	Frequent to constant quivering chin, clenched jaw.
**Legs**	Normal position or relaxed	Uneasy, restless, tense.	Kicking, or legs drawn up.
**Activity**	Lying quietly, normal position moves easily	Squirming, shifting back and forth, tense.	Arched, rigid or jerking.
**Cry**	No cry, (awake or asleep)	Moans or whimpers; occasional complaint	Crying steadily screams or sobs, frequent complaints.
**Consolability**	Content, relaxed.	Reassured by occasional touching hugging or being talked to, distractible.	Difficulty to console or comfort.

Adapted from The FLACC: A behavioral scale for scoring postoperative pain in young children, by S Merkel and others, 1997, Pediatr Nurse 23(3), p. 293–297. Copyright 1997 by Jannetti Co. University of Michigan Medical Center.

Because pediatric pain in young infants is so difficult to clearly identify, we felt that it would be prudent to use other secondary outcome measures such as crying time and heart rate. Both of these measures have been widely used in the neonatal studies on sucrose efficacy for procedural pain, and crying time has been used as the primary outcome measure in many [2,4], and [5]. Crying time was monitored by a stopwatch from the infant's first cry after venipuncture and recorded as the number of seconds that vocalizations were sustained, up to 5 minutes. From previous studies of similar procedures, the majority, but not all infants ceased to cry within three-minutes [15]. Heart rate was measured pre procedure and at 1 minute intervals after the procedure for 5 minutes. The heart rate outcome measure is the difference between the highest value recorded over that 5-minute period and the baseline measure recorded prior to the procedure. We alighted upon this method of assessing heart rate from a review of previous studies in this area as it is commonly used as an outcome measure and seemed reasonable and similar in concept to those previous studies [5]. For the FLACC score, the outcome measure is the difference between pre and post procedure.

### Data collection

All patients aged 0 – 6 months who arrived during study hours were identified in the PED, and the research nurse was contacted. The research nurse recruited and followed patients if eligible. Eligible patients were those meeting inclusion and exclusion criteria. The research nurse explained the study to the family, obtained written consent and gathered some baseline information about the subjects' past medical history. Patients were randomly assigned to one of four groups as follows: a) sucrose b) sucrose & pacifier c) placebo d) placebo & pacifier. Each child received, by mouth, 2 ml of either 44% sucrose or sterile water, two minutes prior to venipuncture. These solutions were prepared and coded in advance by pharmacy such that all other study participants and investigators were blinded to their identity.

Timers were used by the research nurse to coordinate all of the following events. The solution was administered by the research nurse to the anterior aspect of the tongue over 30 seconds via syringe and a pacifier was inserted orally according to randomization. At 2 minutes after commencement of solution administration, venipuncture took place as performed by the PED nurses and as per standard nursing practice. Parents interacted with voice or touch as per normal.

The research nurse collected all data. Baseline vitals such as temperature, weight and BP were recorded. A baseline pain score pre- venipuncture was assigned by the research nurse using the FLACC scale. Each child had continuous cardiac and oxygen saturation monitoring throughout the intervention and data collection. Heart rate and oxygen saturation were noted each minute over a 5-minute period post venipuncture. Crying time post venipuncture was measured by stopwatch. The research nurse assigned a FLACC score between 30 seconds and one minute after the procedure. If initial venipuncture was unsuccessful, a second attempt only took place after the full 5-minute interval. All data was recorded on a data collection sheet and was entered into a spreadsheet by the research nurse for analysis. The research nurse called each participant within 72 hours to assess for adverse effects. All documentation was locked in a secure cabinet, kept confidential for the length of the study and will be destroyed in five years. Data was entered into Microsoft Excel by the research nurse. The data was later downloaded into S- Plus where the majority of the data-analysis was done.

### Data analysis

#### Sample size

Based on previous measurements of pain on the Premature Infant Pain Profile (PIPP- a 20 point scale), we estimated that the standard deviation of pain scores on the FLACC scale (a 10 point scale) to be approximately 1.75. Assuming an alpha level of 0.05 and a power of 90%, we required a total sample size of 84 infants to be able to detect a 1.25 -point average FLACC scale difference between two groups using a paired t-test. Sample size was based on our primary variable of interest and primary outcome (sucrose/pacifier effect on pain reduction) and calculated using nQuery Advisor version 4.0. Although pacifier/sucrose interaction effect was unknown we assumed no interaction in making this calculation.

### Statistical analysis

A two-way analysis of variance was used to ascertain any interaction effects between our primary variables. Differences in intervention groups were computed both in unadjusted (via unpaired t-tests) and adjusted (via regression analysis) analyses for all continuous outcomes of interest (i.e. FLACC change score, crying time, and maximum heart rate difference). For the adjusted analysis, co-variates included in the regression were age, sex, weight, NPO time, and gestational age. The gestational ages for nine children were unavailable, and thus the mean of the remainder of the children was used to impute a value for these nine for purposes of the regression analysis.

The intention to treat principle was used in all our analyses – all subjects were analyzed in the groups to which they were initially assigned. Means, standard deviations, and/or 95% confidence intervals are presented for all continuous outcomes. P-values of statistical tests are presented for all outcomes.

## Results

Over two 3-month periods from February 2004 – June 2005, 87 patients were assessed for eligibility and 84 were randomized to the four groups. Timing of recruitment was dependent on research nurse availability, patient volume and cost. Two parents refused to participate and one patient was deemed too ill to engage in the study. Baseline characteristics of subjects in all groups are presented in Table 2. Most of the baseline characteristics were similar and did not differ between study periods. The most notable difference was that by chance the pacifier & placebo group had a mean and median age that was half of the other three groups. This group also had the highest admission rate. The baseline FLACC score and heart rates were similar between the groups. None of the babies were crying before venipuncture occurred. All of the subjects had successful initial IV attempts.

**Table 2:** Mean clinical and demographic characteristics of the four groups

**Mean Values (Standard Deviation)**	**Sucrose N = 21**	**Placebo N = 19**	**Pacifier & Sucrose N = 22**	**Pacifier & Placebo N = 22**
**Age (days)**	63 (71)	64 (55)	68 (61)	38 (39)
**Weight (kg)**	4.7 (1.8)	4.9 (1.4)	4.9 (1.2)	4.2 (1.1)
**Gest Age (wks)**	37 (3.9)	39 (1.3)	39 (2.9)	38 (2.1)
**NPO (min)**	43 (36)	43 (60)	73 (169)	42 (50)
**Rectal Temp (°C)**	37 (1.0)	37 (0.9)	37 (0.6)	37 (0.8)
**O2 Sat (%)**	97 (2.0)	96 (2.3)	97 (2.0)	97 (2.8)
**Resp Rate (bpm)**	40 (8.5)	40 (10.7)	40 (10.6)	40 (10.4)
**Male Sex**	11	13	11	9
**Admitted**	13	12	14	17
**Previous Analgesia**	0	2	5	2

Table 3 presents the means and 95% confidence intervals of the outcome variables by the four study groups. The ANOVA did not show any signs of an interaction effect between pacifier and sucrose with respect to any of the outcomes examined, thus direct t-test comparisons could be used to ascertain the effects of both sucrose and pacifier using the full sample.

**Table 3:** Mean variables and 95% confidence intervals of the four groups

	**Sucrose**	**Placebo**	**Pacifier & Sucrose**	**Pacifier & Placebo**
**N =**	21	19	22	22

**FLACC Difference**	3.71 (1.58, 5.84)	4.84 (2.80, 6.88)	2.64 (0.88, 4.40)	2.45 (0.92, 3.98)
**Crying Time (sec)**	209.1 (162.1, 255.2)	251.2 (215.8, 286.7)	129.6 (80.7, 178.4)	157.0 (115.9, 198.1)
**Heart rate (bpm)**	19.6 (9.1, 30.2)	28.2 (20.3, 36.0)	36.2 (17.7, 46.7)	24.9 (15.6, 34.2)

### Unadjusted effects

Estimates for FLACC score and heart rate are presented as mean change from baseline ± standard deviation.

#### Sucrose

The FLACC score in the 43 sucrose infants increased from baseline by an average of 3.2 ± 3.6 which was not significantly different from the 3.6 ± 3.3 average of the 41 placebo infants (p = 0.66). There were also no significant differences in crying time (sucrose: 168.4 ± 112.2, placebo: 200.7 ± 96.0; p = 0.16) or heart rate change (sucrose: 28.1 ± 29.3, placebo: 26.4 ± 18.7; p = 0.75).

#### Pacifier

The 40 infants that did not receive a pacifier had an average increase from baseline in FLACC score of 4.3 ± 4.5 compared to the average increase from baseline of 2.5 ± 3.7 in the 44 infants that did receive a pacifier. The difference between the two groups was very close to being statistically significant (p = 0.06). The difference in crying time between the two groups was statistically significant (pacifier: 143.3 ± 101.7, no pacifier: 229.1 ± 90.6; p = 0.0001) while the difference in heart rate change was not (pacifier: 30.6 ± 27.7; no pacifier: 23.7 ± 20.4; p = 0.20).

### Adjusted effects

We ran a regression analysis on our three outcomes (FLACC difference, crying time, and heart rate difference) including our two interventions, as well as age, sex, weight, NPO time, and gestational age.

For change in FLACC score, age was the only variable that was found to significantly affect the pain score (older children experienced more pain, p = 0.006). Neither sucrose nor pacifier was found to significantly affect FLACC score change.

It was a different story with crying time, as both sucrose (sucrose group 47.6 ± 18.8 seconds less crying time than placebo; p = 0.01) and pacifier (pacifier group 80.5 ± 18.7 seconds less crying time than no pacifier; p < 0.0001) had significant effects. Crying time increased with both increasing age (older children cried longer, p < 0.0001) and increasing gestational age (children with higher gestational age cried longer, p = 0.02). This was estimated such that every week of age gain resulted in crying 8.5 seconds longer.

For change in heart rate, none of our co-variates had a significant effect.

### Subgroup analysis

The results of the regression analysis prompted us to do a post-hoc subgroup analysis, stratifying by age. We divided the children into three subgroups: 0–1 month, 1–3 months, and 3–6 months (Tables 4 and 5).

**Table 4:** Differences between sucrose and placebo with 95% confidence intervals

	**n**	**Change in FLACC score**	**Crying Time (seconds)**	**Change in heart rate (bpm)**
**Total Sample**				

**Unadjusted**	84	-0.40 (-2.20, 1.41)	-32.3 (-77.7, 13.1)	1.7 (9.0, -12.4)
**Adjusted**	84	-0.86 (-2.50, 0.78)	-47.6 (-84.5, -10.7)	0.9 (-9.8, 11.6)

**Unadjusted Results by Age Groups**				

**0–1 month**	36	-1.22 (-3.50, 1.06)	-52.7 (-117.5, 12.2)	-3.6 (-16.7, 9.6)
**1–3 months**	28	-1.66 (-5.27, 1.96)	-52.2 (-142.1, 37.6)	4.6 (-20.2, 29.3)
**3–6 months**	20	1.50 (-1.82, 4.82)	6.3 (-61.9, 74.5)	6.9 (-14.3, 28.0)

**Table 5:** Differences between pacifier and no pacifier with 95% confidence intervals

	**n**	**Change in FLACC score**	**Crying Time (seconds)**	**Change in heart rate (bpm)**
**Total Sample**				

**Unadjusted**	84	-1.70 (-3.47, 0.07)	-85.8 (-127.8, -43.8)	6.9 (-3.8, 17.5)
**Adjusted**	84	-1.44 (-3.08, 0.20)	-80.5 (-117.1, -43.9)	8.1 (-2.7, 18.9)

**Unadjusted Results by Age Groups**				

**0–1 month**	36	-1.77 (-4.01, 0.47)	-76.5 (-138.6, -14.5)	9.3 (-3.6, 22.1)
**1–3 months**	28	-2.08 (-5.66, 1.50)	-123.9 (-201.6, -46.3)	0.9 (-23.9, 25.7)
**3–6 months**	20	-0.70 (-4.02, 2.62)	-42.4 (-105.9, 21.1)	11.2 (-9.0, 31.4)

Due to the small sample sizes in this analysis, none of the subgroups showed a statistically significant difference for either pacifier or sucrose with respect to change from baseline in FLACC score, although interestingly both interventions showed much greater effect in the 0–1 month and 1–3 month groups than the older than 3 month group.

For crying time, the sucrose intervention was not significant in any of the three groups, but again showed greater improvement in the younger two groups. Crying time was significantly reduced for pacifier versus non-pacifier in both the 0–1 month and 1–3 month groups, despite the small sample sizes. Subgroup analysis revealed a mean crying time difference of 76.52 seconds (p < 0.0171) (0–1 month) and 123.9 seconds (p < 0.0029) (1–3 month). For subgroup age > 3 months pacifier did not have any significant effect on crying time.

### Other

The only adverse event that was noted was one episode of vomiting which occurred in a total of three children, one in each of the groups except for the sucrose only group.

## Discussion

Our results suggest that venipuncture is a procedure that causes moderate pain in infants. A FLACC score increase of 4.84 (placebo group), post venipuncture falls into the rating of moderate pain as per the authors of the FLACC scale [17-19].

Currently the standard practice during venipuncture in young infants in the PED is not to administer any analgesia. Even though neonatal studies have previously demonstrated the effectiveness of sucrose and/or pacifiers, this practice has not been adopted in general in emergency departments as well as other pediatric departments [20]. We hope that this study will demonstrate the ease of use of sucrose and/or pacifier and we hope that this will inspire practice change in this area.

Our choices of outcome measures were a result of a review of the literature. It should be noted that without direct verbal corroboration from the infants we cannot be entirely sure that any of the above outcome measures actually reflect degree of pain. Previous studies have relied on assessments of behavioral and physiological changes as indirect indicators of pain. We felt that the most comprehensive approach was to use a combination of a validated pain scale, total crying time and change in heart rate. The FLACC scale uses parameters similar to many of the neonatal pain scales, is highly reliable, has been validated, is very easy to use and teach and was best suited to the age group we wished to study. Although crying is associated with pain, it is not exclusive to pain, and thus must be interpreted with caution. In this study, none of the infants were crying prior to the procedure and all cried after it, so it is likely that the pain of this procedure induced this behavioral response. Thus we feel that in this study, crying time is a reasonable measure of pain or discomfort and have interpreted the results in such a light.

Our results show that pacifier appears to be an effective analgesic for the procedural pain of venipuncture in infants. Even though statistical significance was only narrowly missed for the primary outcome measure (p= 0.06), a change in average FLACC score from 4.3 (no pacifier) to 2.5 (pacifier) would be considered by most to be of clinical significance. Pacifier use significantly reduced crying time (statistically and clinically), particularly in the 0–3 month age group, despite small sample sizes of sub-group analysis. It is promising to see that this analgesic effect seems to extend beyond the neonatal period, perhaps up to three months of age. It appears that the effect wanes with age beyond three months. Further trials with larger sample sizes in this age group would be helpful to clarify this matter however.

One caution in the interpretation of results surrounding pacifier use was the fact that the observer was not blinded. This presents potential bias that was unavoidable for the primary outcome measure assessment, as it was necessary to look closely at the infants' faces to give a rating to this parameter on the FLACC scale. The addition of a second observer for the outcome measure of crying time would nearly have doubled the budget of our study and was thus impractical in our setting.

Also, heart rate measurements were assessed at the minute marks only and it is possible that these data points do not accurately represent interim variabilities. This may explain why differences in heart rate were not found. Another possibility is that heart rate monitoring may not be a reliable indicator of the amount of pain experienced. One adult study observed a decrease in heart rate in some patients, likely due to vagal stimulation, on insertion of an IV [21]. Two adult observational studies have noted lack of correlation of heart rate with pain or changes in pain intensity [21,22]. Review of neonatal studies reveals that heart rate data collection methodology is highly variable and there often does seem to be dissociation between pain scale findings and physiological responses such as heart rate [5,23]. Pereira et al evaluated the validity of heart rate measurements for neonatal pain assessment in an RCT and concluded that heart rate variations are an inconsistent and insensitive way to evaluate pain in that population [24]. Further clarification as to the reliability of this outcome measure as an indicator of pain across the pediatric spectrum may be warranted.

For sucrose as analgesia, the results are less clear. T-test results demonstrated no significant benefit; however age adjusted regression analysis showed significant reduction in crying time. Trends seem to show greater reduction in the younger age sub-groups. Sucrose appears to be less effective with increasing age at the dosage studied. Further study with larger sample sizes and perhaps using stronger concentrations of sucrose would be required to determine the upper age limit for the effectiveness of sucrose. It seems that sucrose and pacifier have an additive beneficial effect when used together and perhaps this is where the best use for sucrose as analgesia lies- to be used in conjunction with pacifier.

One must consider the dose of sucrose used. We chose 0.88 g (2 ml of a 44% solution) as this was easily prepared by our pharmacy, which uses an 88% sucrose solution to mix oral pediatric medications, and diluted this solution for the purposes of our study. Doses up to 0.5 g have been studied and determined to be safe for use in the neonatal period [5] and immunization studies have used doses as high as 2.5 g for older infants [14,16] without adverse events. Future studies could look closely at the issue of optimal doses, especially with older infants.

There are several limitations to our study. One limitation to our study was that the study population was a convenience sample of patients and a few potentially eligible patients were not enrolled. The research nurses were available for 8–16 hours during the day so some children arriving overnight may have been missed. It is unlikely, however that these children would have been different from our study population.

Another limitation of this study was that despite accurate randomization, our randomization produced somewhat of an "unlucky sample" in that there were imbalances in some of the baseline statistics particularly age, NPO, and rate admitted. NPO was found in our adjusted analyses to not have an effect on our outcomes, while admission rates were not too unbalanced, and would be unlikely to have an effect on our final outcomes. Due to our determination in the adjusted analysis that, older children tend to experience more pain, the lower age in the pacifier/placebo group could lead to slight overestimation of pain relief in pacifiers and an underestimation of pain relief in the sucrose. These results may need to be interpreted with caution.

The intention of this study was to recruit infants between the ages of 0 and 6 months. Although infants across this entire age spectrum were recruited, numbers at the upper end of this range were less than had been desired, reflecting the visit and illness spectrum of this group and also chance (Tables 4 and 5). For the 84 infants recruited, the median age was 48 days, the mean age was 30 days and only 20 infants fell into the 3–6 month age range. Thus younger infants were represented strongly and older infants were underrepresented in this study. Therefore we could not draw valid conclusions about the effectiveness of our interventions on infants older than 3 months of age.

We also observed a higher standard deviation than we had originally anticipated. As a result, statistical significance was not achieved when examining age related effects although intriguing trends towards significance were seen which warrant further examination.

## Conclusion

This study demonstrates that venipuncture in infants is a moderately painful procedure. The use of pacifier with sucrose as procedural analgesia for venipuncture in the PED is effective in reduction of pain in infants 0–3 months old, as shown by decrease in crying times. Pacifiers and sucrose are inexpensive, easy to use, have quick onset, short duration of action, and no serious side effects. They should be used in the pediatric emergency department and other pediatric units to help prevent pain from venipuncture for infants aged 0–3 months. Further study to clarify effects of age and sucrose concentrations, as well as effectiveness for other painful procedures is required.

## Abbreviations

FLACC, Faces Legs Activity Cry and Consolability; NICU, Neonatal Intensive Care Unit; PED, Pediatric Emergency Department; NPO, Nil per Os; ANOVA, Analysis of Variance, SCH, Stollery Children's Hospital; PO, per Os;

## Competing interests

The author(s) declare that they have no competing interests.

## Authors' contributions

SC, HJ, TK, SA conceived the study. SC, HJ and TK designed the study. SC and TK obtained research funding. SC supervised the data collection, data analysis. BV carried out the statistical analysis and contributed significantly to manuscript write-up of those sections. SC wrote up protocols, grant applications and manuscript. Authors HJ and SA contributed significantly to critical revision of those documents. All authors read and approved the final manuscript. SC takes responsibility for the paper as a whole.

## Pre-publication history

The pre-publication history for this paper can be accessed here:



## References

[B1] Anand KJS, the International Evidence-Based Group for Neonatal Pain (2001). Consensus statement for the prevention and management of pain in the newborn. Archives of Pediatrics & Adolescent Medicine.

[B2] Stevens Bonnie (1999). Pain in infants. Pain; Clinical Manual.

[B3] Franck LS, Greenberg CS, Stevens B (2000). Pain Assessment in Infants and Children. Pediatric Clinics of North America.

[B4] The American Academy of Pediatrics and the American Pain Society (2001). The Assessment and Management of Acute Pain in Infants, Children, and Adolescents. Pediatrics.

[B5] Stevens B, Yamada J, Ohlsson A (2004). Sucrose for analgesia in newborn infants undergoing painful procedures. The Cochrane Database of Systematic Reviews.

[B6] Blass EM, Hoffmeyer LB (1991). Sucrose as an analgesic for newborn infants. Pediatrics.

[B7] Blass EM, Watt LB (1999). Suckling- and sucrose-induced analgesia in human newborns. Pain.

[B8] Ramenghi LA, Evans DJ, Levene MI (1999). "Sucrose analgesia": absorptive mechanism or taste perception?. Archives of Disease in Childhood Fetal & Neonatal Edition.

[B9] Gradin M, Schollin J (2005). The role of endogenous opioids in mediating pain reduction by orally administered glucose among newborns. Pediatrics.

[B10] Eriksson M, Finnström O (2004). Can daily repeated doses of orally administered glucose induce tolerance when given for neonatal pain relief?. Acta Paediatrica.

[B11] Thyr M, Sundholm Ai, Teeland L, Rahm V (2007). Oral glucose as an analgesic to reduce infant distress following immunization at the age of 3, 5 and 12 months. Acta Paediatrica.

[B12] Allen KD, White DD, Walburn JN (1996). Sucrose as an analgesic agent for infants during immunization injections. Archives of Pediatrics & Adolescent Medicine.

[B13] Barr RG, Young SN, Wright JH, Cassidy KL, Hendricks L, Bedard Y, Yaremko J, Leduc D, Treherne S (1995). "Sucrose analgesia" and diphtheria-tetanus-pertussis immunizations at 2 and 4 months. Journal of Developmental & Behavioral Pediatrics.

[B14] Lewindon PJ, Harkness L, Lewindon N (1998). Randomized controlled trial of sucrose by mouth for the relief of infant crying after immunization. Archives of Disease in Childhood.

[B15] Ramenghi LA, Webb AV, Shevlin PM, Green M, Evans DJ, Levene MI (2002). Intra-oral administration of sweet-tasting substances and infants' crying response to immunization: A randomized, placebo-controlled trial. Biology of the Neonate.

[B16] Reis EC, Roth EK, Syphan JL, Tarbell SE, Holubkov R (2003). Effective pain Reduction for Multiple Immunization Injections in Young Infants. Archives of Pediatrics and Adolescent Medicine.

[B17] Voepel-Lewis T, Merkel SI, Tait AR, Trzcinka ABS, Malviya S (2002). The reliability and Validity of the Faces Legs, Activity, Cry and Consolability observational tool as a measurement of pain in children with cognitive impairment. Anesthesia and Analgesia.

[B18] Willis MH, Merkel SI, Voepel-Lewis T, Malviya S (2003). FLACC behavioral pain assessment scale: a comparison with the child's self-report. Pediatric Nursing.

[B19] Merkel SI, Voepel-Lewis, Shayevitz JR, Malviya S (1997). The FLACC: A Behavioral Scale for Scoring Postoperative Pain in Young Children. Pediatric Nursing.

[B20] MacLean S, Obispo J, Young K (2007). The Gap Between Procedural Pain Management Treatments Available and Actual Practice. Pediatric Emergency Care.

[B21] Bartfield J, Janikas J, Lee R (2003). Heart rate response to intravenous catheter placement. Acad Emerg Med.

[B22] Bossart P, Fosnocht D, Swanson E (2007). Changes in Heart Rate Do Not Correlate With Changes in Pain Intensity in Emergency Department Patients. The Journal of Emergency Medicine.

[B23] Anand KJS (2007). Pain Assessment in Preterm Neonates. Pediatrics.

[B24] Pereira AL, Guinsburg R, de Almeida MFB, Montiero AC, dos Santos AMN, Kopelman BI (1999). Validity of behavioral and physiologic parameters for acute pain assessment of term newborn infants. São Paulo medical journal.

